# Alkhurma Hemorrhagic Fever in Humans, Najran, Saudi Arabia

**DOI:** 10.3201/eid1612.100417

**Published:** 2010-12

**Authors:** Abdullah G. Alzahrani, Hassan M. Al Shaiban, Mohammad A. Al Mazroa, Osama Al-Hayani, Adam MacNeil, Pierre E. Rollin, Ziad A. Memish

**Affiliations:** Author affiliations: Ministry of Health, Riyadh, Saudi Arabia (A.G. Alzahrani, H.M. Al Shaiban, M.A. Al Mazroa, O. Al-Hayani, Z.A. Memish);; Centers for Disease Control and Prevention, Atlanta, Georgia, USA (A. MacNeil, P.E. Rollin)

**Keywords:** Alkhurma virus, Alkhurma hemorrhagic fever, Flavivirus, Saudi Arabia, hemorrhagic fever, tickborne, livestock, viruses, zoonoses, research

## Abstract

Alkhurma virus is a flavivirus, discovered in 1994 in a person who died of hemorrhagic fever after slaughtering a sheep from the city of Alkhurma, Saudi Arabia. Since then, several cases of Alkhurma hemorrhagic fever (ALKHF), with fatality rates up to 25%, have been documented. From January 1, 2006, through April 1, 2009, active disease surveillance and serologic testing of household contacts identified ALKHF in 28 persons in Najran, Saudi Arabia. For epidemiologic comparison, serologic testing of household and neighborhood controls identified 65 serologically negative persons. Among ALKHF patients, 11 were hospitalized and 17 had subclinical infection. Univariate analysis indicated that the following were associated with Alkhurma virus infection: contact with domestic animals, feeding and slaughtering animals, handling raw meat products, drinking unpasteurized milk, and being bitten by a tick. After multivariate modeling, the following associations remained significant: animal contact, neighboring farms, and tick bites.

## Abstract 2

TOC summary: Infection was associated with tick bites and contact with farm animals.

Alkhurma virus (ALKV) was discovered in Saudi Arabia in 1995 in a butcher with suspected Crimean-Congo hemorrhagic fever. His fever developed after he had slaughtered a sheep from the city of Alkhurma. Diagnostic testing identified a flavivirus as the etiologic agent (*1**,**2*). Subsequently, ALKV was isolated from the blood of 6 male butchers in Jeddah, and another 4 cases were diagnosed serologically. This disease was named Alkhurma hemorrhagic fever (ALKHF) because the first case was reported from the Alkhurma governorate (*1*).

After initial virus identification, from 2001 through 2003, another 37 suspected ALKHF cases, of which 20 were laboratory confirmed, were reported in Alkhumra district, south of Jeddah (*3*). Among the 20 patients with confirmed cases, 11 had hemorrhagic manifestations and 5 died.

Full genome sequencing has indicated that ALKV is a distinct variant of Kyasanur Forest disease virus, a virus endemic to the state of Karnataka, India (*4*). Recently, ALKV was found by reverse transcription–PCR in *Ornithodoros savignyi* ticks collected from camels and camel resting places in 3 locations in western Saudi Arabia (*5*). ALKHF is thought to be a zoonotic disease, and reservoir hosts may include camels and sheep. Suggested routes of transmission are contamination of a skin wound with blood of an infected vertebrate, bite of an infected tick, or drinking of unpasteurized, contaminated milk (*6*).

Several studies have been conducted to describe the characteristics and determinants of ALKHF (*1**,**3**,**5**,**6*). We conducted a case–control study to assess associated risk factors.

## Materials and Methods

### Study Area

The study was conducted in the city of Najran, which is in the southern part of Saudi Arabia on the border with Yemen. It is the capital of Najran region and has a population of ≈250,000. It is an agricultural city in which residents commonly raise domestic animals in their backyards. Cases of ALKHF were found in 6 districts, which were close to each other (within ≈30 km) and rural and in which hygiene was poor.

### Case Identification

From 2006 through 2009, laboratory testing for ALKV was performed for Najran residents who sought medical care and whose illnesses met the case definition for suspected ALKHF. Infection with ALKV was suspected if a patient had acute febrile illness for at least 2 days; negative Rift Valley fever, Crimean-Congo hemorrhagic fever, and dengue confirmatory test results; and >2 of the following: 1) at least 3-fold elevation of alanine transferase or aspartate transferase or clinical jaundice; 2) signs of encephalitis such as confusion, disorientation, drowsiness, coma, neck stiffness, hemiparesis, paraparesis, or convulsions; 3) signs of hemorrhage such as ecchymosis, purpura, petechiae, gastrointestinal bleeding (hematemesis, melena, hematochesia), epistaxis, bleeding from puncture sites, or menorrhagia; and 4) platelet count <100 × 10^9^/L, or lactate dehydrogenase or creatine phosphokinase 2× upper reference level.

In addition, as part of public health surveillance, blood samples were collected from household contacts of patients with laboratory-confirmed ALKHF. Samples from persons seeking medical care were tested by ELISA for ALKV-specific immunoglobulin (Ig) M and IgG by using ALKV antigen as described (*7**,**8*) and for viral-specific sequence by reverse transcription–PCR (TiBMolbiol, LightMix kit; Roche Applied Science, Basel, Switzerland). Samples from follow-up testing of household contacts were tested by ELISA for ALKV-specific IgG. A total of 11 cases were identified through persons seeking medical care whose illnesses met the case definition for ALKHF, and another 17 cases were identified through follow-up testing of household contacts.

### Case–Control Study

A case of ALKHF was defined as illness in any person who lived in the catchment area of Najran General Directorate of Health Affairs and who had serologic evidence of ALKV infection during January 1, 2006–April 30, 2009. All 28 case-patients identified were included in the study. For each case-patient, 2 controls selected from the same house or the nearest neighboring house. A total of 65 controls were enrolled, each of whom was serologically negative for ALKV-specific antibodies.

A structured questionnaire was based on information collected during the initial review of the outbreak and asked for demographic data (name, age, gender, nationality, educational status, occupation, marital status, and place of residency), clinical features, and possible risk factors such as exposure to domestic animals. Exposure history for case-patients was limited to the 30 days before onset of illness and for controls to the past 30 days. If the case-patient or control was a child and unable to respond, the child’s mother, father, or an older family member helped answer the questions.

### Data Analysis

After the samples (frequency and percentage distributions, means, and standard deviations) were described, cross-tabulations were constructed to compare risk factors. To estimate the strength of association, we calculated odds ratios (ORs) with 95% confidence intervals (CIs). For the multivariate analysis, the most significant variables from the bivariate analysis (at p<0.05) and relevant variables thought to be associated with ALKHF from other studies were selected for backward stepwise inclusion in the model.

## Results

### Outbreak Description

Of 28 case-patients, 11 (39%) were seeking medical advice and hospitalized for ALKHF, and the others were identified through surveillance among household contacts. Among the 11 hospitalized case-patients, 7 diagnoses of ALKHF were made by PCR and 4 by serologic testing. Among the hospitalized case-patients, only 1 had severe symptoms and was admitted to the intensive care unit. The remaining 10 stayed in the hospital for 5–15 days (mean duration 9.3 ± 3.3 days) and received supportive care, including intravenous fluid administration and antimicrobial drugs when indicated.

Although only 11 case-patients sought medical care, 4 reported having had an illness during the study period with symptoms consistent with those of ALKV infection, and 13 seropositive persons reported having had no illness. Among all seropositive persons, fever was found for 15 (54%), bleeding (epistaxis) for 8 (29%), rash for 7 (25%), change in urine color for 6 (21%), gum bleeding for 5 (18%), neurologic signs (e.g., neck rigidity) for 3 (11%), and change in feces color for 1 (4%) (Table 1). No case-patient died. Timing of seeking care for the 11 case-patients was December 2006 (n = 1), January 2007 (n = 1), 2008 (n = 4), and the first 4 months of 2009 (n = 5) (Figure); most cases occurred during March–July. Among the 28 persons with positive serologic test results, 5 clusters were identified in which multiple persons within a single family were infected (these family clusters involved 7, 5, 3, 3, and 2 persons in the respective families).

**Table 1:** Clinical signs of Alkhurma hemorrhagic fever virus among 28 patients, Najran, Saudi Arabia, 2006–2009

Clinical feature	No. (%) patients		
	Hospitalized, n = 11	Not hospitalized, n = 17	Total
Fever	11 (100)	4 (24)	15 (54)
Nosebleed	5 (46)	3 (18)	8 (29)
Rash	5 (46)	2 (12)	7 (25)
Jaundice	5 (46)	1 (6)	6 (21)
Change in urine color	5 (46)	1 (6)	6 (21)
Gum bleeding	5 (46)	0	5 (18)
Neck rigidity	3 (27)	0	3 (11)
Numbness of extremities	2 (18)	0	2 (7)
Seizures	2 (18)	0	2 (7)
Change in feces color	1 (9)	0	1 (4)

**Figure:**
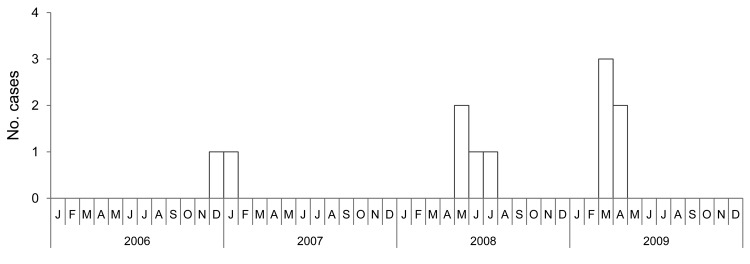
Annual distribution of Alkhurma hemorrhagic fever case-patients admitted to hospitals in Najran, Saudi Arabia, 2006–2009.

The 28 case-patients were 4–55 years of age (mean 22 ± 11.2 years), and most (14 [50%]) were 20–39 years of age. Most case-patients were male (18 [64%]) and single (15 [54%]). Case-patients were of 3 nationalities: Yemeni (24 [86%]), Saudi (3 [11%]), and Bangladeshi (1 [4%]). Regarding educational level, 1 (4%) was in preschool, 4 (14%) were illiterate, 13 (46%) had primary or intermediate degree, 8 (29%) had a high school degree, and 2 (7%) had a university degree. Most (12 [43%]) case-patients were students, 4 (14%) had livestock-related jobs, 5 (18%) were housewives, and 7 (25%) had other jobs. Most (20 [71%]) case-patients lived in modern houses (Table 2).

**Table 2:** Demographic characteristics of persons with and without Alkhurma hemorrhagic fever, Najran, Saudi Arabia, 2006–2009

Characteristic	Case-patients, no. (%), n = 28	Controls, no. (%), n = 65	Total, no. (%), n = 93	OR (95% CI)
Age, y				
<20	13 (46)	24 (37)	37 (40)	5.42 (0.58–125.74)
20–39	14 (50)	31 (48)	45 (48)	4.52 (0.50–10.43)
>40	1 (4)	10 (15)	11 (12)	Reference
Gender				
M	18 (64)	31 (48)	49 (53)	1.97 (0.72–5.45)
F	10 (36)	34 (52)	44 (47)	Ref
Marital status				
Single	15 (54)	30 (46)	45 (48)	1.35 (0.51—3.63)
Married	13 (46)	35 (54)	48 (52)	Reference
Nationality				
Saudi	3 (11)	14 (22)	17 (18)	0.86 (0.05–28.00)
Yemeni	24 (86)	47 (72)	71 (76)	2.04 (0.19–50.75)
Bangladeshi	1 (4)	4 (6)	5 (5)	Reference
Education				
Preschool	1 (4)	6 (9)	7 (8)	0.25 (0.01–6.51)
None (illiterate)	4 (14)	7 (11)	11 (12)	0.86 (0.06–12.21)
Primary	9 (32)	29 (45)	38 (41)	0.85 (0.18–4.15)
Intermediate	4 (14)	11 (17)	15 (16)	0.55 (0.04–7.15)
Secondary	8 (29)	9 (14)	17 (18)	1.33 (0.12–15.74)
University	2 (7)	3 (5)	5 (5)	Reference
Occupation				
Livestock related†	4 (14)	9 (14)	13 (14)	1.27 (0.23–6.79)
Student	12 (43)	17 (26)	29 (31)	2.02 (0.57–7.34)
Housewife	5 (18)	19 (29)	24 (26)	0.75 (0.17–3.30)
Other‡	7 (25)	20 (31)	27 (29)	Reference
District				
Al Hadhan	4 (14)	5 (8)	9 (10)	2.80 (0.25–36.19)
Al Balad	7 (25)	15 (23)	22 (24)	1.63 (0.21–15.05)
Al Jarbah	12 (43)	29 (45)	41 (44)	1.45 (0.22–11.83)
Al Mashaliah	3 (11)	9 (14)	12 (13)	1.17 (0.10–14.06)
Al Ghwaila	2 (7)	7 (11)	9 (10)	Reference
House				
Modern	20 (71)	50 (77)	70 (75)	0.75 (0.25–2.30)
Traditional	8 (29)	15 (23)	23 (25)	Reference

*OR, odds ratio; CI, confidence interval. †Shepherd, butcher. ‡Teacher, driver, military, none.

### Outbreak Setting

Cases were identified within 6 different districts; most were in the city of Najran. They were in agricultural areas with livestock, some of which lived with the people in the houses.

### Patient Demographics and Risk Factors

The mean age of case-patients was 22.3 years ± 11.2 years, and mean age of controls was 25.2 years ± 15.4 years; the difference was not significant (*t* test, p = 0.657). The age group 20–39 years contained 14 (50%) case-patients and 31 (48%) controls; the age group <20 years contained 13 (46%) case-patients and 24 (37%) controls. No significant difference was noted between case-patients and controls in terms of gender, nationality, education level, marital status, type of home (modern or not), occupation, or district of residence (Table 2).

Among the case-patients, 14 (50%) gave a history of owning or raising domestic animals, compared with 17 (26%) controls (OR 2.82, 95% CI 1.02–7.91). Among those who reported owning or raising domestic animals, all case-patients and controls had sheep on their farms; thus, owning sheep was a significant risk factor (OR 2.82, 95% CI 1.02–7.91). Owning cows and camels was less common; no significant association was found (OR 1.37, 95% CI 0.30–5.86 and OR 10.29, 0.84–279.2, respectively). Risk for infection was significantly higher for those who lived <100 meters from farms (OR 4.00, 95% CI 1.40–11.75) than for those who lived farther from farms (Table 3).

**Table 3:** Risk factors for Alkhurma hemorrhagic fever virus, Najran, Saudi Arabia, 2006–2009*

Risk factor	Case-patients, no. (%), n = 28	Controls, no. (%), n = 65	Total, no. (%), n = 93	OR (95% CI)
Owning or raising domestic animals				
Yes	14 (50)	17 (26)	31 (33)	2.82 (1.02–7.91)
No	14 (50)	48 (74)	62 (67)	Reference
Owning the following animals				
Sheep	14 (50)	17 (26)	31 (33)	2.82 (1.02–7.91)
Cows	4 (14)	10 (15)	14 (18)	1.37 (0.30–5.86)
Camels	3 (11)	1 (2)	4 (4)	10.29 (0.84–279.20)
None	14 (50)	48 (74)	62 (67)	Reference
Owning animal with abnormalities				
Yes	5 (36)	7 (41)	12 (39)	0.79 (0.14–4.31)
No	9 (64)	10 (59)	19 (61)	Reference
Owning animals with the following type of abnormality				
Sudden death	2 (14)	5 (29)	7 (23)	0.44 (0.04–3.78)
Recurrent abortion	1 (7)	1 (6)	2 (7)	1.11 (0.00–49.10)
Weakness	2 (14)	1 (6)	3 (10)	2.22 (0.12–74.91)
None	9 (64)	10 (59)	19( 61)	Reference
Living how far from farm, meters				
<100	20 (71)	25 (39)	45 (48)	4.00 (1.40–11.75)
>100	8 (29)	40 (62)	48 (52)	Reference
Contact with domestic animals				
Yes	13 (46)	9 (14)	22 (24)	5.39 (1.74–17.30)
No	15 (54)	56 (86)	71 (76)	Reference
Feeding animals				
Yes	9 (32)	3 (5)	12 (13)	9.79 (2.11–51.48)
No	19 (68)	62 (95)	81 (87)	Reference
Milking animals				
Yes	7 (25)	5 (8)	12 (13)	4.00 (0.99–16.64)
No	21 (75)	60 (92)	81 (87)	Reference
Slaughtering animals				
Yes	10 (36)	6 (9)	16 (17)	5.46 (1.54–20.02)
No	18 (64)	59 (91)	77 (83)	Reference
Handling raw meat products				
Yes	9 (32)	7 (11)	16 (17)	3.92 (1.14–13.84)
No	19 (68)	58 (89)	77 (83)	Reference
Drinking unpasteurized milk				
Yes	8 (29)	6 (9)	14 (15)	3.93 (1.06–14.88)
No	20 (71)	59 (91)	79 (85)	Reference
Being bitten by tick				
Yes	10 (36)	3 (5)	13 (14)	11.48 (2.51–59.73)
No	18 (64)	62 (95)	80 (86)	Reference
Being bitten by mosquito				
Yes	25 (89)	49 (75)	74 (80)	2.72 (0.65–13.03)
No	3 (11)	16 (25)	19 (20)	Reference

*OR, odds ratio; CI, confidence interval.

A significantly higher proportion of case-patients (46%) than controls (14%) had direct contact with animals (OR 5.39, 95% CI 1.74–17.3). Furthermore, 9 (32%) case-patients and 3 (5%) controls fed animals (OR 9.79, 95% CI 21.1–51.48); 10 (36%) case-patients and 6 (9%) controls slaughtered animals (OR 5.46, 95% CI 1.54–20.02); and 9 (32%) case-patients and 7 (11%) controls handled raw meat products (OR 3.92, 95% CI 1.14–13.84). A borderline significant association with disease was found for milking animals (OR 4.00, 95% CI 0.99–16.64). Risk was higher for those who dealt with animals in multiple ways (e.g., feeding, slaughtering, milking, handling raw meat products) (χ^2^ for trend 15.53; p<0.001). Unpasteurized milk was consumed by 8 (29%) case-patients and 6 (9%) controls (OR 3.93, 95% CI 1.06–14.88). A statistically significant association was found for tick bites and disease; a higher proportion of case-patients (36%) than controls (5%) reported a history of tick bites (OR 11.48, 95% CI 2.51–59.73). No statistically significant difference between case-patients and controls was found for exposure to mosquitoes (OR 2.72, 95% CI 0.65–13.03) (Table 2).

Multivariate analysis, conducted with variables that were significant (p<0.05) in bivariate analysis and with variables previously reported to be associated with ALKHF (contact with animals, tick bites, close proximity of neighboring farms, consumption of unpasteurized milk, and mosquitoes bites) was conducted by backward stepwise regression analysis. Among these variables, contact with animals, tick bites, and neighboring farms remained predictors for ALKHF (adjusted ORs 3.17, 95% CI 0.96–10.43; 6.20, 95% CI 1.34–28.70; and 3.63, 95% CI 1.25–10.4, respectively) (Table 4).

**Table 4:** Multivariate logistic regression results of risk factors for Alkhurma hemorrhagic, Najran, Saudi Arabia, 2006–2009*

Risk factor	Crude OR (95% CI)	Model aOR† (95% CI)
Contact with domestic animals	5.39 (1.74–17.3)	3.17 (0.96–10.43)
Tick bites	11.48 (2.51–59.73)	6.20 (1.34–28.70)
Adjacent farm distance	4.00 (1.40–11.75)	3.63 (1.25–10.49)

*OR, odds ratio; CI, confidence interval; aOR, adjusted OR. †aOR for risk factors (contact with domestic animals, tick bites, adjacent farm distance) after elimination of nonsignificant variables (drinking unpasteurized milk and owning or raising domestic animals) calculated by using backward stepwise strategy.

## Discussion

Our findings that some patients had subclinical illness and that no deaths were documented among the 28 case-patients suggest that previous studies may not have characterized the full spectrum of ALKV-associated illness and that case-fatality rates as high as 25% may have resulted from detecting only severe cases of ALKHF. In addition, our identification of multiple seropositive members within families suggests the occurrence of family-based clusters of ALKV infection.

ALKV-positive persons with subclinical disease, identified by the active surveillance system, did not undergo thorough clinical and laboratory investigations and were not directly observed by a clinician. Because of this lack of observation and because of the inability of some case-patients to recall and report low-grade fever within 1 month before onset of illness, only 53% of case-patients reported fever, but all case-patients who were hospitalized had fever. Our findings underscore the role of the high percentage of case-patients with subclinical infection in the epidemiology of this disease; seroprevalence studies should be encouraged.

A similar seasonal pattern of disease (March–July) was found in western provinces (Jeddah and Makkah) among 11 case-patients who recovered during 1994–1999. This finding might support evidence of disease association with the peak activity of ticks in the beginning of March (*9**,**10*).

Similar to other hemorrhagic fevers, ALKHF showed no predilection for patient age, gender, or nationality (*11*). Risk factors identified by this study included a broad array of activities associated with animal exposures but most significantly with direct contact with animals. Similarly, a higher proportion of case-patients than controls owned or raised animals; however, no difference was noted in the proportion reporting abnormalities in their animals. This finding might suggest the low virulence of the virus in animals and highlights the need for animal studies. Among the animals raised, sheep were significantly associated with the disease.

Although we found no significant association between ALKHF infection and livestock-related occupations such as butchering, we found a high association for history of slaughtering livestock. These findings agree with those of other studies conducted in the cities of Jeddah and Makkah (*1**,**3*). Furthermore, we found that another major risk factor for human infection was direct contact with blood or body fluids from animals.

Ingestion of unpasteurized milk has been noted as a risk factor in previous studies; the mode of transmission is not yet clear but has been suggested to result from contamination of the milk (*6**,**12*). However, our multivariate analysis found no association.

Only 10 of the 28 interviewed case-patients had a history of a tick bite within 1 month before symptom onset; however, tick exposure was significantly more common among case-patients than among controls. The association of tick bites and ALKV was supported by Charrel et al., who detected ALKV DNA in ticks (*Ornithodoros* spp.) collected from camels and camel resting places in western Saudi Arabia (*5*).

ALKV has been identified only in the southern and western parts of Saudi Arabia. Given our current evidence that subclinical ALKV infections occur in humans, the virus may be more widespread in Saudi Arabia than previously realized. We are conducting studies to further characterize the distribution of ALKV in Saudi Arabia. In addition, the history of the reported disease in Makkah during the Hajj, when thousands of livestock are imported to Saudi Arabia, and the existence of the outbreak in Najran, which is at the border with Yemen, necessitate further studies in adjacent countries (*3**,**5*). Additionally, ALKV is closely related to Kyasanur Forest disease virus, which has been well characterized in India. The possibility remains that this virus has a wider geographic range in the Middle East and central Asia than previously realized.

Because our investigations were retrospective, we cannot exclude recall bias about exposure, but as long as the controls were from the same households, they were exposed to some of the questions during the surveillance done by the preventive department. Furthermore, the investigators who administered the questionnaires based the questions on the month before the interview, which should minimize recall bias.

ALKHF is a zoonotic disease with clinical features ranging from subclinical asymptomatic to severe complications. This study highlights the different activities related to exposure to animals and tick bites in the transmission of ALKV to humans; it found no significant association with mosquitoes. Further studies are needed to understand the role of livestock, wildlife, and ticks in the maintenance of the virus and the risk factors so that public health measures can be developed to reduce the extent of the disease in humans.
